# Recurrent Bloodstream Infections Without Sepsis in a Patient With Short Bowel Syndrome on Parenteral Nutrition: A Case of Potential Sepsis Tolerance

**DOI:** 10.7759/cureus.89601

**Published:** 2025-08-08

**Authors:** Akiva Brin, Sarah Israel, Sigal Matza-Porges, Zvi Ackerman

**Affiliations:** 1 Medicine, Hadassah-Hebrew University Medical Center, Mount Scopus Campus and the Hebrew University-Hadassah Medical School, Jerusalem, ISR; 2 Microbiology and Infectious Diseases, Hadassah-Hebrew University Medical Center, Mount Scopus Campus and the Hebrew University-Hadassah Medical School, Jerusalem, ISR; 3 Human Genetics, Institute for Medical Research, The Hebrew University of Jerusalem, Jerusalem, ISR

**Keywords:** bacteremia recurrent, short bowel syndrome, tolerance to endotoxin, tolerance to sepsis, total parenteral nutrition

## Abstract

Adults with short bowel syndrome (SBS), malabsorption, and malnutrition often require long-term parenteral nutrition (PN), typically as total PN (TPN). These patients are susceptible to bloodstream infections and sepsis. We present a case of a 63-year-old male patient who developed SBS following an acute mesenteric event. During his follow-up, he experienced recurrent episodes of bacteremia and fungemia, likely due to non-adherence to infection prevention guidelines. Surprisingly, all but the final episode did not progress to sepsis. He recovered from these infections with fluid resuscitation and appropriate systemic antibiotics or antifungals and removal of the infected venous access when indicated. We hypothesize that this patient developed a state of tolerance to sepsis. The potential mechanisms that contribute to protection from sepsis in this patient with recurrent bloodstream infections are discussed.

## Introduction

Adults with short bowel syndrome (SBS) can experience various complications, including diarrhea resulting from diverse pathophysiological mechanisms and malabsorption of macro- and micronutrients leading to deficiencies in electrolytes (such as magnesium, potassium, calcium, and bicarbonate), fat-soluble vitamins, essential fatty acids, and trace elements (including iron, zinc, and selenium) [1]. Furthermore, these patients are prone to increased fluid loss within the intestinal lumen, potentially leading to chronic dehydration. Consequently, they may require long-term parenteral nutrition (PN), typically as total PN (TPN), and fluid replacement via indwelling central venous catheters [1]. Adult SBS patients are prone to develop venous catheter malfunction, bloodstream infections, and central vein thrombosis. Bloodstream infection in patients with SBS may present with signs of sepsis, like fever, chills, and hemodynamic instability [1, 2]. In order to prevent central line-associated bloodstream infection and its complications, certain infection control measures should be practiced [3]. 

In this report, we describe a patient with SBS who experienced recurrent episodes of bacteremia and one episode of fungemia, most likely due to non-adherence to instructions for preventing bloodstream infections. Unexpectedly, all but the last of these episodes did not develop into sepsis. The potential mechanisms contributing to tolerance to bloodstream infections in SBS patients receiving PN are discussed [4-12].

## Case presentation

A 63-year-old man (weight 60 kg, height 170 cm, BMI 20.8 kg/m²) presented in May 2015 with a severe ischemic mesenteric event. His past medical history included long-term smoking, type II diabetes mellitus, and hyperlipidemia. During a prolonged hospitalization, he underwent several surgical interventions, including an extended right hemicolectomy, partial resection of the jejunum and ileum, and cholecystectomy, ultimately resulting in an end-jejunostomy and colonic mucous fistula. A primary workup for a hypercoagulable state was negative. Postoperatively, the patient developed signs of severe malnutrition with deficiencies primarily in vitamins B12, A, D, E, and K, along with chronic hypokalemia, hypocalcemia, hypomagnesemia, dehydration, and progressive renal failure. His weight dropped to 42 kg (BMI 14.5 kg/m²). TPN and daily infusions of six to 10 liters of intravenous fluids and electrolyte solutions were initiated through a central venous access. A few months later, he could tolerate a limited amount of oral solid and liquid intake. However, he still required high-volume PN and intravenous fluid infusions. A course of daily teduglutide administration was started, enabling weaning from PN a few months later. However, due to persistent high-volume stoma effluent, he continuously required high-volume infusions of fluids and electrolyte solutions. PN weaning was sustained for only a few months, contingent upon the patient's adherence to teduglutide administration. 

During the eight and a half years from May 2015 to December 2022, the patient was admitted multiple times to various departments (including emergency, internal medicine, surgical, and day-care) of two tertiary medical centers in our city. The indications for these recurrent admissions included complaints such as "not feeling well," episodes of high fever, abdominal pain, suspected dehydration, severe hypomagnesemia or hypokalemia, and multiple mechanical issues with the central venous lines and the abdominal wall stoma. Notably, based on our experience with this patient, the medical team routinely drew blood cultures from the central line and/or a peripheral vein during each of his numerous referrals, regardless of his symptoms. Over this period, 42 episodes of bacteremia and two episodes of fungemia were documented. In 19 of these episodes, bacterial and fungal organisms were recovered from two or more sets of blood cultures drawn simultaneously from both central and peripheral veins. Two of the bacteremia episodes were polymicrobial (Table 1). The broad spectrum of pathogens likely represents contamination from the abdominal wall stoma, mouth, skin, and hospital environment. It was believed that these repeated episodes of bacteremia and fungemia were primarily due to non-adherence to instructions for preventing bloodstream infections. During periods when a caregiver responsible for handling his intravenous solutions was available, no episodes of bacteremia or fungemia were reported. All bloodstream infection episodes (except the last one) resolved within a few days following the administration of fluids and appropriate systemic antibiotics or antifungals and removal of the infected venous access when indicated. During all these episodes, no systemic signs of sepsis, such as hemodynamic or respiratory instability, new altered mentation, or high fever, were observed. Furthermore, there was no need for vasopressors or mechanical respiratory support. The hospital course of the patient's last episode of bloodstream infection in December 2022 differed from the previous episodes. The patient was readmitted with *Klebsiella pneumoniae* extended-spectrum beta-lactamase bacteremia. During this hospitalization, he developed signs of sepsis with hemodynamic and respiratory instability and showed no response to full supportive care, including appropriate antibiotics, vasopressors, and mechanical respiratory support.

**Table 1:** List of infective agents that were recovered from two or more blood culture sets that were concomitantly withdrawn, from both central and peripheral veins. ESBL: extended-spectrum beta-lactamase; MRSA: methicillin-resistant *Staphylococcus aureus*; MSSA: methicillin-susceptible *Staphylococcus aureus*, *co-infection with *Staphylococcus haemolyticus*; **co-infection with *Streptococcus mitis*

Organism	Polymicrobial bloodstream infection	Number of positive blood culture sets in each episode
Acinetobacter ursingii	No	3
Candida glabrata	No	3
Enterobacter cloacae complex	No	4
Enterobacter cloacae complex	No	3
Klebsiella oxytoca	No	4
Klebsiella pneumoniae	No	2
Klebsiella pneumoniae	No	2
Klebsiella pneumoniae	No	4
*Klebsiella pneumoniae *(ESBL)	No	4
*Klebsiella pneumoniae* (ESBL)	No	5
Pseudomonas aeruginosa	No	4
Pseudomonas oryzihabitans	No	5
*Staphylococcus aureus *(MRSA)	No	3
*Staphylococcus aureus *(MRSA)	No	7
*Staphylococcus aureus *(MSSA)	No	7
Staphylococcus epidermidis	No	3
Staphylococcus epidermidis	No	5
*Staphylococcus aureus* (MRSA)	Yes*	4
Staphylococcus haemolyticus	Yes**	2

## Discussion

Our patient had several comorbidities before the acute ischemic mesenteric event. After his recovery from the surgical procedures, he was diagnosed with SBS, malnutrition with depletion of minerals and trace elements, progressive chronic renal failure, and the need for chronic administration of TPN/PN and high-volume intravenous fluid and electrolyte solutions. Patients with SBS on chronic TPN are prone to developing immune system impairment, as well as recurrent bloodstream infections and sepsis [1-6]. Moreover, patients who have experienced sepsis and concurrent malnutrition have been found to have a poor prognosis; a significant correlation has been observed between the severity of objective indices of malnutrition and 30-day mortality [2,6]. 

Alterations and impairments of the immune system, particularly peripheral T-cell function, have been reported in adult patients with SBS treated with TPN [4]. These defects include impaired proliferative response to mitogens and reduced tumor necrosis factor-alpha (TNF-α) production in response to anti-CD3 monoclonal antibodies in peripheral blood lymphocytes (PBL). Furthermore, the expression of the T-cell receptor, involved in signal transduction and subsequent T-cell activation, was found to be downregulated [4]. It has been suggested that these defects in PBL function may be secondary to the extensive resection of gut-associated lymphoid tissue due to massive small intestinal resection [4]. Our patient experienced multiple episodes of bloodstream infections without any clinical evidence of sepsis, except for the final episode. Sepsis, unlike bloodstream infection, is defined as an infection that triggers a maladaptive host response and organ dysfunction [7]. The pathogenesis of sepsis is complex, involving not only the infectious process and the initial host response but also the activation of various pro- and anti-inflammatory cytokines, activation of the coagulation cascade and the complement system, immune suppression (including apoptosis of various immune cell types), mitochondrial dysfunction, and alterations in the microbiome [7,8]. Inflammation is initiated to eradicate the infectious agent and varies in intensity depending on the pathogen type, load, and virulence, as well as the patient's comorbidities, immune status, sex, age, and nutritional status. Anti-inflammatory responses regulate inflammation and facilitate repair mechanisms while contributing to homeostasis [7,8]. 

The occurrence of repeated episodes of Gram-negative bacteremia in children with SBS has been previously reported [5]. Similar to our patient, Hajam et al. reported that some children with SBS exhibited only modest symptoms during episodes of Gram-negative bacteremia. These authors hypothesized that plasma from SBS children contains various protective factors that prevent deterioration during *Escherichia coli* (*E. coli*) bacteremia. They demonstrated that plasma from these children inhibited the production of both TNF-α and interleukin-6 induced by either *E. coli *or lipopolysaccharide (LPS)-stimulated host cells. Furthermore, mice treated intravenously with plasma samples from SBS or healthy subjects showed reduced pro-inflammatory cytokine levels in plasma and a significant survival advantage after *E. coli* infection. The authors suggested that SBS children may be tolerant to sepsis-like situations due to the presence of microbe-specific antibodies, impairment of T-cell responses, or a combination of both [5]. A similar phenomenon of better outcomes among TPN patients with staphylococcal bacteremia was reported by Gompelman et al. [9]. This group evaluated the clinical characteristics and outcomes of 620 episodes of *Staphylococcus aureus* bacteremia in two patient groups: 53 episodes in patients receiving chronic TPN for intestinal failure and 567 episodes in patients not receiving TPN.

The authors of this study assumed that the portal of entry in 91% of staphylococcal bacteremia episodes in the TPN patients was a central venous catheter and that the mode of acquisition was either healthcare-associated (83%) or hospital-acquired (17%). Compared to the non-TPN patients, TPN patients exhibited significantly lower levels of C-reactive protein and fewer events of persistent fever during the first 72 hours after the start of antibiotics. Moreover, evaluation revealed that TPN patients had a more favorable prognosis than non-TPN patients; the overall mortality during a six-month follow-up period after the first positive blood culture was significantly lower (10% vs. 34%) [9]. The authors of this study suggested that the more favorable course of staphylococcal bacteremia in TPN patients might be attributed to their characteristics: they were younger, had a higher percentage of females, and had fewer comorbidities. Additionally, these patients were likely more aware of their risk of bloodstream infection and therefore promptly contacted their medical providers at the first signs of infection [9]. Herein, we propose two additional hypotheses for the better prognosis of bloodstream infections in patients receiving chronic TPN: the presence of either tolerance to sepsis or a genetic polymorphism in a gene encoding a molecule involved in the sepsis cascade. 

For the past two decades, a new concept of tolerance to disease, particularly sepsis tolerance, has been investigated [7,8]. Disease tolerance does not directly affect the microbial pathogen load. Instead, it minimizes the host's vulnerability to cell and tissue damage caused by microbial pathogens and/or the immune response against them. The exact mechanisms inducing disease tolerance remain elusive; however, recent publications reporting data from experimental animals and humans have highlighted the critical role of molecular and cellular components of the innate and adaptive immune systems [7,8]. An endotoxin-tolerant state is characterized by a significant reduction in the inflammatory capacity of innate immune cells, such as monocytes/macrophages, upon subsequent endotoxin challenge. The induction of tolerance to endotoxin in circulating monocytes of healthy subjects has been previously reported. del Fresno et al. reported that tolerance induction in monocytes occurred after one hour of LPS exposure. These monocytes became tolerant to further LPS exposure for a limited period of five days, after which they regained their pro-inflammatory responsiveness [10]. Draisma et al. reported that five days after intravenous administration of low-dose LPS to healthy subjects, their monocytes exhibited a state of endotoxin tolerance [11]. The host's response to an invading pathogen, as well as susceptibility to sepsis and prognosis, may depend on the host's genetic background, particularly genetic variants in immune genes involved in the sepsis process. Recently published epidemiological studies suggest that certain single-nucleotide polymorphisms in genes encoding TNF-α or pro- and anti-inflammatory cytokines involved in the sepsis and septic shock cascade, as well as the coagulation system, may influence the course and outcome of sepsis [12]. To investigate the possibility that genetic polymorphisms or pathogenic variants were involved in the endotoxin tolerance observed in our patient, whole-exome sequencing was planned. However, informed consent for genetic testing was not obtained. 

In summary, the patient described herein had a very unusual clinical course. It may be assumed that he suffered from multiple bloodstream infections due to non-adherence to instructions for preventing bloodstream infections and also due to immune deficiency secondary to the presence of SBS. The state of immune deficiency had probably a dual effect in this patient: on one side, it increased his susceptibility to bloodstream infections, but on the other prevented the progression of bloodstream infections into sepsis. In addition, it may be speculated that he had, in addition, a genetic polymorphism in a gene or genes involved in the sepsis and septic shock cascade. Such polymorphism could also be involved in the unusual ability of this patient to prevent the progression of bloodstream infections into sepsis.

## Conclusions

Patients with SBS receiving TPN may experience recurrent episodes of bacteremia and fungemia. These patients may develop a state of tolerance to sepsis. The potential mechanisms contributing to this tolerance to bloodstream infections in these patients remain unknown. Patients tolerant to sepsis may present with mild clinical complaints and an atypical clinical picture during a bloodstream infection episode. This can mislead the medical team following the patient and may delay the diagnosis and treatment of the bloodstream infection. Therefore, physicians caring for TPN patients should be aware of the possibility of unusual presentations of bloodstream infection in this population. Moreover, it should be remembered that tolerance to sepsis may not be complete or permanent, and these patients remain susceptible to developing potentially fatal clinical sepsis. Every effort should be made to encourage these patients to adhere to measures for preventing bloodstream infections.
